# Influence of different timeframes of antibiotic application on postoperative infections in patients with caesarean section

**DOI:** 10.3389/fsurg.2025.1626402

**Published:** 2026-01-12

**Authors:** Carolin Anhalt, Maurice Kappelmeyer, Georgia Cole, Angela Koeninger, Edith Reuschel

**Affiliations:** Department of Obstetrics and Gynecology, Clinic St. Hedwig of the Order of St. John, University of Regensburg, Regensburg, Germany

**Keywords:** Caesarean section, postoperative infection, postpartum infection, prophylactic antibiotics, timeframe of antibiotic application, wound infection

## Abstract

**Background/objectives:**

Prophylactic, intravenous antibiotics are a known protective factor for surgery-related infections in patients undergoing caesarean section. This study aims to determine the impact of the timing of antibiotics and their influence on postoperative infection-related morbidity. Application 30 min before laparotomy was compared with application after umbilical cord clamping.

**Methods:**

This study retrospectively analyzed the data of 6,034 patients giving birth by Caesarean section in University Clinic St. Hedwig, Regensburg. Germany. 3,001 cases (2017–2019) received a single shot of antibiotics 30 min before skin incision (Group 1), whereas in 3,033 women delivering by Caesarean section (2021–2023) the antibiotic was applied after cord clamping and child development (Group 2). Excluded were 62 cases for showing signs of infection before surgery or having a premature rupture of membranes prior to developing an infection.

**Results:**

20 patients in each group developed surgery-related infections. The calculated Odds Ratio did not differ between groups. The risk for postoperative infection after Caesarean section was 1.6%.

**Conclusions:**

In this study there was not found a significant difference between the two examined time points of antibiotic application in the numbers of postoperative infections. The results did not show an increased maternal risk for surgery-related infections by antibiotic application after cord clamping.

## 1 Introduction

For various reasons worldwide almost 20% of all newborns are delivered by Cesarean section (CS), making it one of the most frequently performed surgeries (1). The risk of postpartum infection is increased five- to twenty-fold, compared to vaginal delivery (2–4). Therefore, as standard procedure a prophylactic single shot antibiotic is administered intravenously, which reduces infectious morbidity by 60%–70% (5). A Cochrane meta-analysis from 2021 suggests that 1st and 2nd generation cephalosporins are as effective as broad-spectrum penicillin plus beta-lactamase inhibitors, so the choice of antibiotic should be made individually (6).

**Figure 1:**
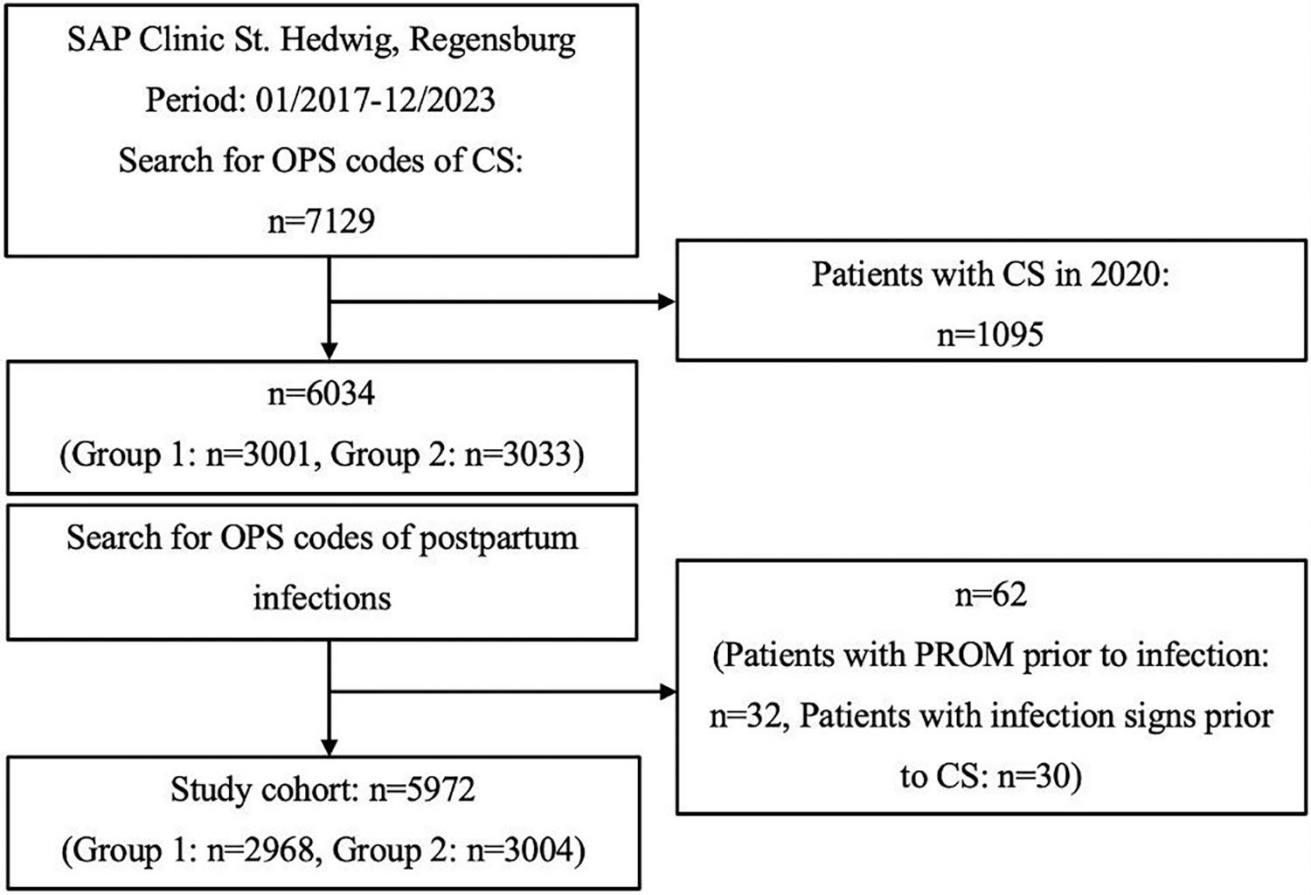
Flowchart of patients included. CS, Caesarean section; PROM, premature rupture of membranes.

**Figure 2:**
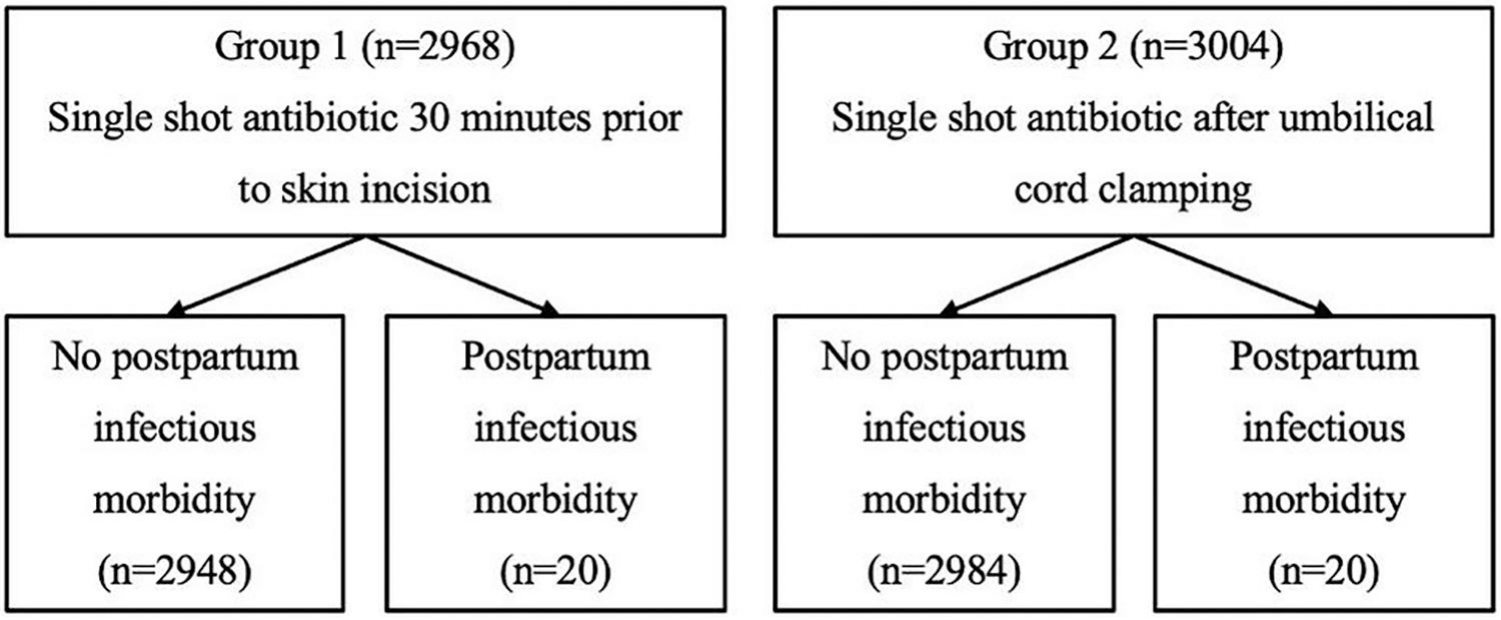
Visual representation of study results.

While the use of prophylactic antibiotics in patients undergoing CS is undisputed, the timing of administration is still subject of discussions (7).

Current guidelines endorse the administration of antibiotics within 60 min before skin incision (SI) to allow a therapeutic concentration to build up in the mother's tissue (8–11). This is based on findings of meta-analyses and systematic reviews, that showed a significant reduction of the risk for composite maternal infections, when antibiotics were given within 60 min to SI compared to after umbilical cord clamping (CC) (1, 3, 12). The benefit was proven to be significant in endometritis (1, 3, 12) and wound infection (1, 3).

A potential disadvantage of antibiotic application before CC is allowing the antibiotics to reach fetal circulation through the placenta (13). The antibiotics will be cleared within 24 h from the neonate's blood with the half-life of Cefuroxime being three times longer as in adults (13). As the adverse effects on the newborn have not yet been fully investigated (14), the possible consequences of prebirth applied antibiotics cannot be easily estimated.

This gives reason to reevaluate whether antibiotics should be given before SI and thereby reach the fetus. A large-scale research study was done in 75 Swiss hospitals to examine whether the results of meta-analyses and reviews could be applied to Switzerland as a country with high health standards (7). In conclusion, contrary to the meta-analyses (1, 3, 12), no disadvantage of antibiotic administration after CC could be found compared to prior to SI (7). Taking the results of Sommerstein et al. into consideration, we can assume a similar result in Germany.

As this could be of substantial benefit for newborns and possibly challenges the guidelines, conducting a German study concerning this matter was of great importance. This study was performed in the Clinic St. Hedwig of The Order of St. John, University of Regensburg, as a monocentric study, which is one of its main strengths. Therefore, increased comparability between both cohorts was achieved as conditions did not vary, except for the timing of antibiotics' administration. To further control influencing factors amongst the groups, strict exclusion criteria were developed. The purpose was to exclude cases of prenatal infections, such as patients with premature rupture of membranes (PROM), who have an elevated risk for postpartum infection.

## 2 Materials and methods

For this case-control-study, data of the University Department of Obstetrics and Gynecology, Clinic St. Hedwig of The Order of St. John, University of Regensburg, Germany, was retrospectively analyzed. Visual illustration of the process of study design can be found in Figure 1 and Figure 2. The study was conducted in accordance with the Declaration of Helsinki and approved by the Ethics Committee of the University of Regensburg (protocol code 25-4138-104, date of approval: 14.04.2025). The clinical software SAP extracted a list of all cases of CS between 2017 and 2023 using OPS Codes for elective and unplanned CS (*n* = 7,129). The OPS classification is the official German encoding system for surgical procedures and medical interventions (15). As a standard Cefuroxime 1.5 gram (intravenously) was used as the prophylactic antibiotic agent. The cases were divided into two groups, whereby in the period from 2017 to 2019 the prophylactic single shot antibiotic was given 30 min before SI (Group 1), and from 2021 to 2023 the antibiotic dose was administered after the development of the child and CC (Group 2). Cases from 2020 (*n* = 1,095) were excluded as the timing of antibiotic application was changed within that year. This resulted in a total number of 6,034 patients, with 3,001 cases in Group 1 and 3,033 in Group 2.

Further analysis in the clinical software was conducted, using OPS codes for postpartum infections, wound infections, puerperal and postpartum fever, endometritis, postpartum sepsis, dehiscence and hematoma of the incision and other non-classified postpartum complications attributed to infection. The resulting list was used to individually examine each case for possible surgery-related infection.

Strict exclusion criteria were applied to eliminate any cause of postpartum infection rather than the surgery itself. Women who had a PROM, antibiotic administration during or before labor or an intrapartum CS, prior to developing a postpartum infection, were excluded. Ascertained maternal or fetal signs of infection, like elevated temperature (>37.5 °C) of the mother, signs of intraamniotic infection and laboratory values, showing bacterial infection led to an exclusion from the study. Signs of intraamniotic infection were maternal fever and leukocytosis, fetal tachycardia and purulent cervical discharge (16).

62 cases of postpartum infection were rejected from the study, due to one of the previously mentioned reasons. Among those, 32 patients were excluded due to a PROM prior to developing a postpartum infection, and 30 cases for showing signs of prenatal infection.

5,972 patients were included, of whom 2,968 belong to Group 1 and 3,004 to Group 2. The number of mothers who developed surgery-related infections after CS until discharge or readmission with signs of infection within 30 days was evaluated.

Different criteria were attributed to postpartum and surgery-related infections such as elevated temperature (>37.5 °C), and laboratory values such as elevated c-reactive protein (CRP, >5 mg/L (17)), elevated leukocytes and signs of bacterial infection. Categories, in which the cases were divided, were wound or surgery-site infection (SSI), endometritis, vaginal infection, postpartum fever, sepsis, and urinary tract infection (UTI).

To categorize postpartum morbidity as SSI clinical findings like fever, redness, pain, cloudy fluid discharge or inflammation of the incision had to be present, or bacterial colonization in the smear of the wound area (18). Merely hematoma or dehiscence of the wound were not categorized as infectious morbidity. Acute endometritis is characterized as an infection with foul smelling lochia, elevated production of vaginal discharge, generalized feeling of illness, pelvic pain, and fever (19). Vaginal swabs showing bacterial colonization without general malaise, fever or foul-smelling lochia were not assigned to endometritis but rather vaginal infections.

Temperatures of 38.5 °C and higher were classified as postpartum fever. It is relevant to highlight that each patient was assigned to only one category of postpartum infection. So, a confirmed focus of infection with accompanying fever led to the attribution of the case to one of the previously mentioned categories. Infectious morbidity was categorized as sepsis, if a bloodstream infection with proof of bacteria was documented. UTI were associated with signs of infections and bacteria in the urine.

The program IBM SPSS Statistics Version 29.0.0.0 was used to perform the statistical analysis. Descriptive statistics determined the group sizes and the number of infections in total and groupwise. To explore the effect of different timepoints of antibiotic application the Odds Ratio (OR) was calculated, comparing the groups. Additionally, frequencies of different types of infections were obtained, categorized, and listed.

## 3 Results

### 3.1 Primary outcome

Out of the 5,972 included mothers with CS, 5,932 cases did not develop surgery-related infections, and 40 cases had some sort of postpartum infection. In each group 20 patients developed surgery-related infectious morbidity. This equals 0.67% in Group 1 and 0.67% in Group 2.

So, the overall risk to develop a surgery-related infection after CS in the analyzed cohort is 0.67%. If the excluded cases (cases with signs of infection during or prior to labor, such as peripartum fever, signs of intraamniotic infection or laboratory values, indicating an infection and PROM patients who developed a postpartum infection) are added into the calculation the risk is 1.69%.

### 3.2 Odds ratio

Furthermore, the OR was calculated using a logistic regression model comparing the groups to explore the effect of different timepoints of antibiotic application. The calculated odds ratio (OR = .988) in this study was not statistically significant (*p* = .969, 95% CI.530–1.840). This had to be expected as the data showed the exact number of cases with surgery-related infections in both groups with a similar and high number of cases. Therefore, the results show no advantage of the administration of antibiotics 30 min before SI compared to after CC in reducing surgery-related infections after CS.

### 3.3 Categories of postoperative infectious morbidity

**Figure 3:**
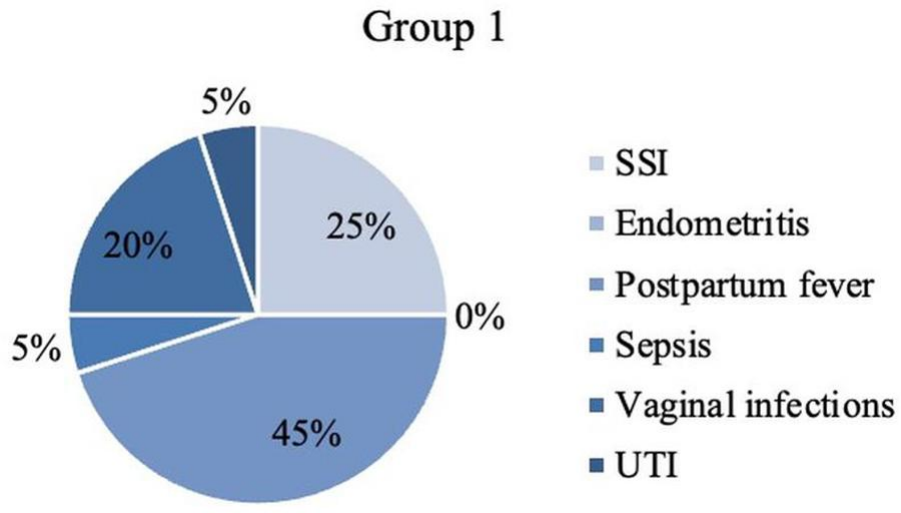
Infectious morbidity in Group 1. SSI, surgical site infection; UTI, urinary tract infection.

**Figure 4:**
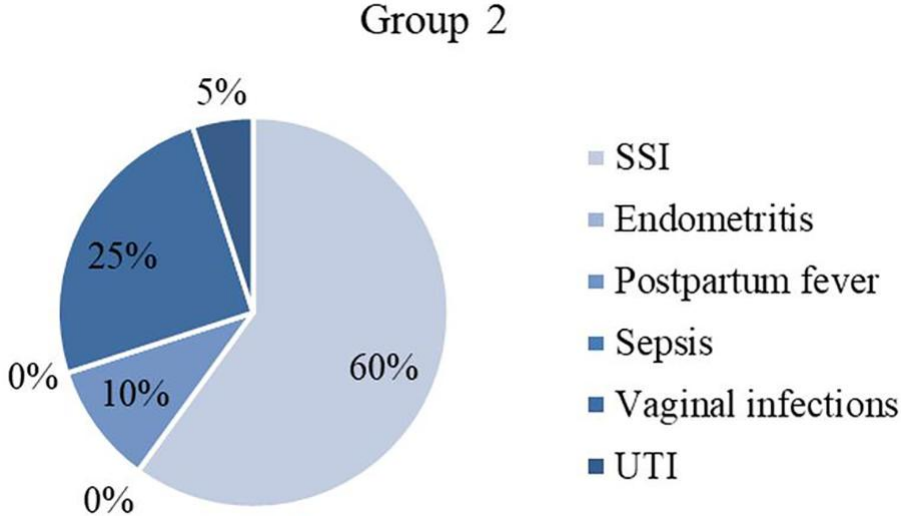
Infectious morbidity in Group 2. SSI, surgical site infection; UTI, urinary tract infection.

**Figure 5:**
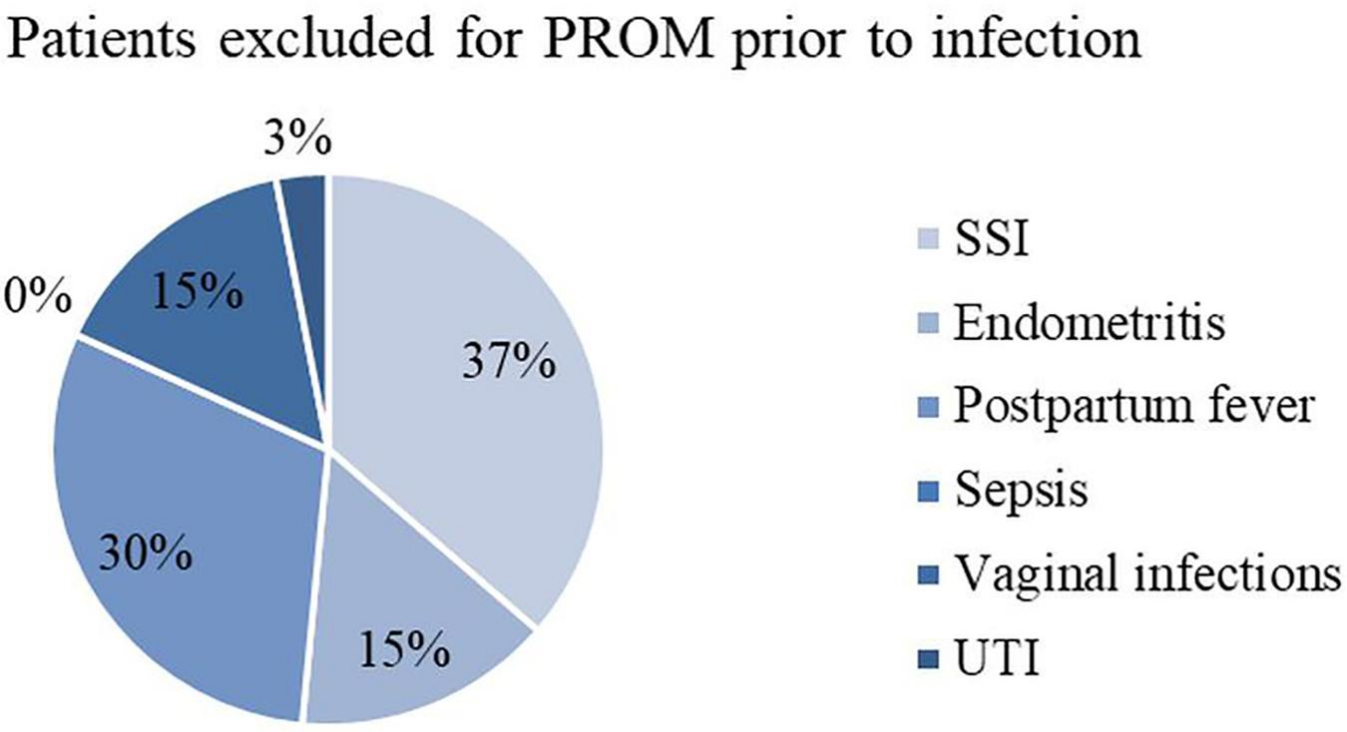
Infectious morbidity in patients excluded for PROM prior to infection. PROM, premature rupture of membranes; SSI, surgical site infection; UTI, urinary tract infection.

**Figure 6:**
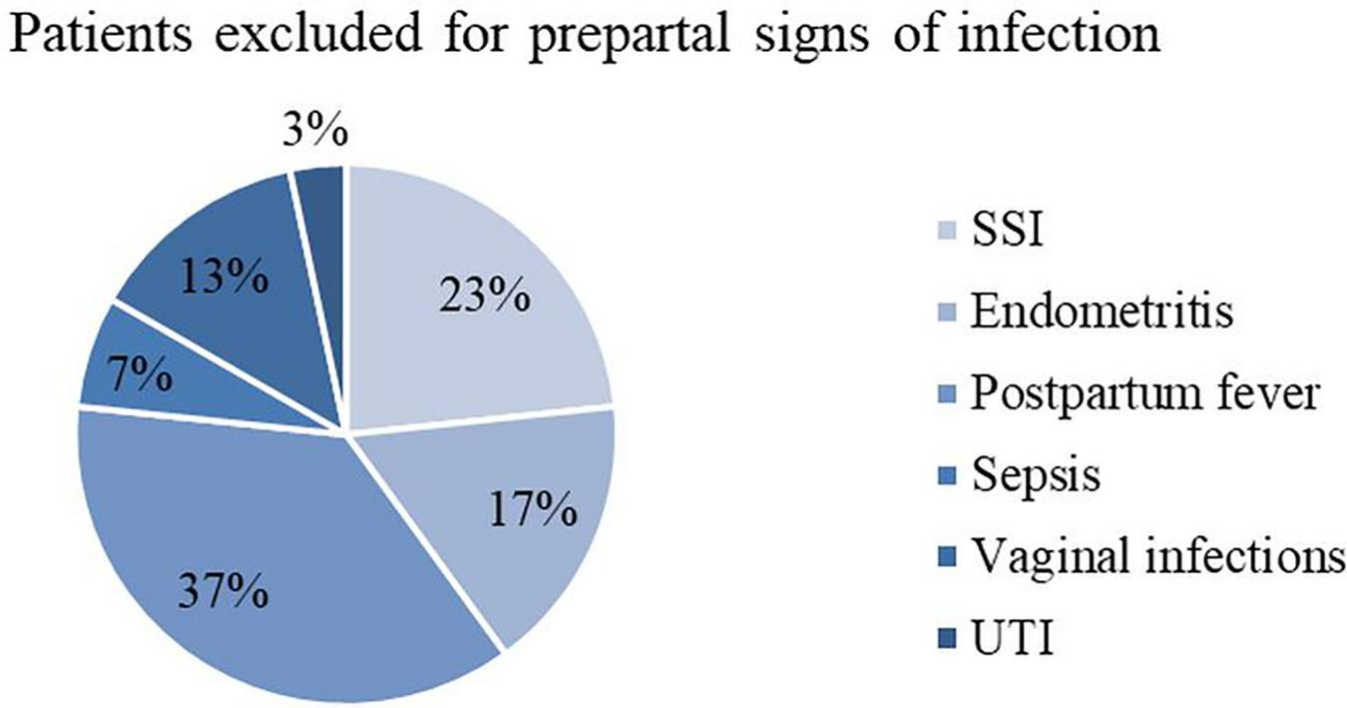
Infectious morbidity in patients excluded for prepartal signs of infection. SSI, surgical site infection; UTI, urinary tract infection.

When having a closer look on postpartum infections, cases are attributed to different categories according to definitions (explained in Material and Methods). Table 1 shows the frequencies of each surgery-related infectious morbidity and Figures 3–6 visually represent the distribution of the different infections in both groups as well as the excluded patients.

**Table 1:** Postoperative infectious morbidity.

Infectious morbidity	Group 1 (*n* = 2,968)	Group 2 (*n* = 3,004)	Patients excluded for PROM prior to infection	Patients excluded for prepartal signs of infection
SSI	5	12	12	7
Endometritis	0	0	5	5
Postpartum fever	9	2	10	11
Sepsis	1	0	2	2
Vaginal infections	4	5	1	4
UTI	1	1	2	1
Total	20	20	32	30

SSI, surgical site infection; UTI, urinary tract infection.

The most remarkable disparity was found in the category endometritis with zero cases in all included patients (*n* = 0) and ten in total over the excluded patients (*n* = 10). Furthermore, it needs to be pointed out that Group 1 showed more patients (*n* = 9) with postpartum fever (group 2: *n* = 2), but less wound infections (SSI) (*n* = 5) than Group 2 (*n* = 12).

## 4 Discussion

This study found no difference in surgery-related postpartum infections between antibiotic application before SI and after CC. A 1.69% risk of postpartum infection after CS over all the cases (including patients with PROM prior to developing an infection and preexisting infection signs) has been detected.

The baseline risk of most meta-analyses concerning this topic differs widely from above mentioned risk (1, 3, 5, 20), showing an average risk of 5.4% or higher (1, 5, 12). One reason for this difference could be the fact that studies from developed and developing countries were included in these studies. The healthcare systems in low-income countries vary substantially from those in well-developed countries, like Germany, and infection and death rates in general are higher (21, 22). Additionally, the spectrum of pathogens differs (1). Previously mentioned reviews unanimously favored the application of prophylactic antibiotics before SI for reduction of composite maternal infectious morbidity (1, 3, 12, 20, 23), endometritis (1, 3, 12, 23–27) and SSI (1, 3, 24). Still, the similarity of results in these meta-analyses is not surprising, as many of the same studies were included (1, 3, 12, 24, 27). It is also important to highlight that these meta-analyses (1, 3, 5, 12) included studies from 1997 (28) and some even earlier (1, 5), which leaves room for questioning whether they might be outdated, as demographics, surgery-prepping techniques and hygiene standards have changed. Sommerstein et al. criticized that some meta-analyses did not account for the fact that the absolute reduction in composite infectious postoperative morbidity was low (3, 7).

In contrast to the mentioned systematic reviews, the results of this study could not detect a benefit of applying antibiotics before CC. This might be due to the low overall risk of surgery-related infections of 1.69%. The timing of prophylactic antibiotic application may not have consequences on the postpartum infectious morbidity, if the infection rate is low, in general. This hypothesis is supported by a large-scale Swiss research study of Sommerstein et al., whose results are applicable to comparable high standard healthcare systems (7). In contrast to most meta-analyses (1, 3, 5, 20), the Swiss overall SSI rate was low (1.7%) and like in this study, they did not find a significant difference between the two timings of antibiotic application (7).

Additional research from high-income countries (Denmark (2, 29), Austria (30), Switzerland (7), USA (31) and China (32) also did not observe an advantage of antibiotic prophylactic application prior to incision, but most of those studies focused on elective CS (29–32). The Danish study of Kuhr et al. investigated antibiotic application after CC in non-elective CS and concluded that this resulted in lower rates of composite infectious morbidity, compared to what was expected (2).

Moreover, the majority (65%) of SSIs found by Sommerstein et al. were only superficial wound infections (7). The severity and treatment options of postpartum infections need to be kept in mind as the prevalence of grave maternal infections is low (2, 29) and women in most cases are treated with oral antibiotics (2).

The strict exclusion of those criteria and the Swiss study (7) might be another reason for the discrepancies to the results of the mentioned meta-analyses. Many of those failed to consequently exclude women with preexisting maternal fever or signs of infections during labor (1, 3, 12, 23–25, 27). In contrast, Sommerstein et al. and this study accounted for preexisting infections and excluded those cases (7). Mothers showing signs of infection before or intrapartum are always at higher risk for postpartum infections. In the analyzed cohort those are treated with antibiotics immediately, instead of waiting until after CC. Furthermore, mothers with PROM show a higher risk of infection (2, 5, 7, 14). The birth channel functions as a site of pathogen-entry (2, 33).

Nevertheless, the absence of adjustment for confounding variables concerning the two cohorts from different time periods (2017–2019 vs. 2021–2023) is the most critical limitation of our study. However, the study was performed at our Clinic St. Hedwig of The Order of St. John, University of Regensburg. The population of both cohorts were pregnant women from Upper Palatinate, Bavaria, who delivered in our hospital. The population characteristics in this part of Bavaria are relatively similar. Also, the staff organization, the perioperative practice and the hygiene protocols did not change notably at our hospital during these two time periods. Only the surgical technique of the cesarean section changed from a continuous uterine suture in 2017–2019 to an interrupted suture from 2021 to 2023.

So, ruling out all possible infection- predisposing factors was essential, as the goal of this study was not to change procedures, as immediate application of antibiotics is obligatory in cases of intrapartum signs of infection or PROM. The purpose was rather to observe whether the application time of antibiotics has an influence on the outcome and, pre-incision antibiotic prophylaxis might not be necessary.

This is of great importance, as the greatest concern of antibiotic application prior to SI are adverse fetal effects (12). The admission of pre-incision antibiotics leads to possibly “unnecessary fetal exposure” by transmission to the neonate via the placenta (12). Therapeutic levels of antibiotics, thereafter, are not only found in the maternal blood, but also in the newborn (14). The antibiotic transfer through the placental barrier presumably differs markedly between individuals, exposing several neonates to adult antibiotic levels (13). Moreover, the median half-life of Cefuroxime is around 3.5 h in newborn, which is about three times longer than in adults (around 70 min) (13). This is suspected to be due to the limited renal function of neonates (13). It is also important to note that some antibiotic transmission will also happen by breastfeeding after CS even if the antibiotic was applied after CC (14). However, by way of breastfeeding the antibiotic reaches the child in subtherapeutic concentrations only (14).

With the antibiotic reaching the newborn's blood circulation and tissues, symptoms of a present infection could possibly be suppressed (7, 12) and resistant strains could increasingly emerge (7, 12, 13). This is a health hazard, as especially in preterms a rise in Ampicillin-resistant Escherichia coli related sepsis has been observed (29). Antibiotics, especially cephalosporins, are known to interact with vitamin K, lowering levels due to impairing its recycling in the liver (34), possibly leading to uncontrollable bleeding (7).

Studies investigated mostly short-term consequences for the child, like newborn sepsis, infection or admission to an intensive care unit: No difference was found to infants whose mothers received the antibiotics after CC (1, 3, 13, 14, 27). It is important to highlight that those studies reported low to moderate quality of evidence and further research is required (1, 14).

The long-term consequences of *in utero* antibiotic exposure to the newborn remain unclear, however. Recent studies have tried to examine its potential impacts on gut microbiota and the development of asthma and allergies (2, 14, 35–37). This is of particular importance as intestinal microbiota in infancy has an impact on health outcomes later in life (14, 38).

The mode of delivery has been proven to influence the composition of the neonate's intestinal bacterial colonization (14, 29, 39). The microbiota of infants born by vaginal delivery varies from those delivered by CS (14). This is not easily attributable to a cause, as CS and antibiotic application usually co-occur and both could be the determining factor. A study by Stearns et al. found similar microbiota in vaginally delivered children, whose mothers received an intrapartum antibiotic treatment, comparing newborn delivered by CS (40). This suggests that antibiotic exposure has an influence on the microbiota independently of mode of delivery (40). The authors stated that in vaginally delivered children, who had received antibiotic exposure during birth (due to GBS), the duration of the antibiotic treatment had an impact (40).

On the contrary, other studies attribute the difference in gut microbiota between vaginally born children and infants delivered by CS to the mode of delivery (14, 41). It remains unclear to which extent antibiotic application before SI influences the fetal microbiota (7, 14, 41).

Intrapartum antibiotics and their impact on intestinal colonization are thought to be related to the development of allergic diseases (12, 14). As the bowel is the “largest immunological tissue in human body” (42), it is not surprising that a correlation between dysbiosis and abnormal immune response has been found (14, 42). Antibiotic treatments in infancy have been linked to asthma, allergies, diabetes type 1, eczema, food allergy and obesity (35–37, 41–45). Moreover, it may be associated with inflammatory bowel diseases, like Crohn's disease (7, 31, 32). Many confounding factors complicate research concerning long-term consequences of antibiotic exposure during labor. Therefore, it is very difficult to link observed effects to their cause.

Without sufficient research on long-term health effects of intrapartum antibiotics in newborn, it seems reasonable to question application before SI and challenge current guidelines. This is especially relevant in countries with good health standards, where no significant difference in the mother's outcome can be found from holding off on antibiotics until after CC (7, 29–32). An appropriate benefit-risk assessment for mother and child needs to be considered (14).

As giving the antibiotic after CC results in acceptable rates of composite infectious morbidity (2), it allows to include patients' wishes in the decision making about the antibiotic timing. A Danish semi-structured interview study questioned fourteen expectant mothers on their choices concerning the timing of antibiotic application (46). After being thoroughly informed about the scientific state, ten out of fourteen women would decide against antibiotics before CC, rather accepting a higher risk of infection than an antibiotic exposure of their newborn (46). This included one patient, who had already had a wound infection before (46). If no elevated risk for mother or child is present and the circumstances allow it (e.g., planned CS), it is appropriate to inform the pregnant women and consider her opinion on the timepoint of antibiotic application.

It also may be sensible to evaluate different parameters in each case before deciding on the timing of antibiotics. There are known risk factors for maternal postsurgical infections especially SSI, which allow a risk stratification for every patient (2, 14, 32). Especially obesity should be considered as it doubles the risk for SSI according to Kuhr et al. (2). Among other factors emergency CS (7, 14, 32), diabetes (2, 14), rupture of membranes (2, 5, 14), hypertension (14), intrapartum fever (2) and preexisting maternal infections (7) seem to elevate the chance of infectious morbidity after CS. So, the internal validity of our study is also impaired by not considering maternal risk factors as well as risk factors for postoperative infections such as mentioned above e.g., BMI, diabetes, hypertensive disorders, smoking, emergency cesarean section, type of anesthesia, duration of labor or maternal fever. In cases where risk factors are present, it should be assessed whether antibiotics prior to SI are expected to improve the outcome. Otherwise, the decision on the antibiotic timing should take the mother's preference into account. Future research regarding long-term effects of intrapartum antibiotics on the newborn is required to adequately balance the risks for mother and child and possibly challenge international guidelines.

This study was not a randomized, double-blinded study but a retrospective case-control-study, so bias cannot be ruled out. An additional limitation is the process of information acquisition. It must be mentioned that there is a very strict coding management at our hospital. Every operation procedure is consistently checked by several coding specialists with their extensive knowledge of medical documentation (e.g., patient treatment), billing systems, and case characterization. Despite that, it must be indicated as a possible weakness of or study that OPS codes for different types of infectious morbidity were used to generate a list of all patients with possible post-surgical infections. Therefore, it is possible that cases were falsely categorized at the respective time. Those were missed in this study, which could possibly explain the low prevalence of composite infectious morbidity, even in comparison with other countries with similar health standards.

Additionally, a further limitation of our study is, that possible mild postoperative infections which may have been treated in outpatient settings e.g., at private practices were probably not be registered. Still, assumably these cases show only mild infections not referring the patient back to the hospital. Furthermore, only cases that developed an infection during their hospital stay or were readmitted to the same hospital have been captured. There was no information about visits to a resident gynecologist or general practitioner of the mothers after discharge. Therefore, mild infectious morbidity after discharge that did not require readmission and was only treated orally was not included in this study.

The main strength of this study is that it was performed as a monocentric study including a very high number of cases. Both cohorts were exposed to relatively similar perioperative and peripartum conditions, which increases comparability. Additionally, the strict exclusion criteria regulated factors that could lead to variation amongst the groups and eliminated possible co-factors. Therefore, our data on the timing of antibiotic prophylaxis in cesarean sections strongly indicate the possibility of administering antibiotic prophylaxis after cord clamping and so protecting the microbiome of the fetus by resolving any possibility of the antibiotic passing into the fetal circulation. The conduction of a multicenter randomized study for more complete data remains to be seen.

## 5 Conclusions

This monocentric retrospective case-control-study does not find benefits in applying antibiotics before SI in comparison to after CC in CS. Based on little evidence on long-term effects on the newborn caused by antibiotics, we advise to avoid antibiotic application before CC. If risk factors for postsurgical infectious morbidity are present, a risk stratification can help to decide whether antibiotics before SI are recommended.

## Data Availability

The datasets for this article are not publicly available due to concerns regarding participant/patient anonymity. Requests to access the datasets should be directed to the corresponding author.
